# The Development of Cooperative Channels Explains the Maturation of Hair Cell’s Mechanotransduction

**DOI:** 10.1016/j.bpj.2019.08.042

**Published:** 2019-09-12

**Authors:** Francesco Gianoli, Thomas Risler, Andrei S. Kozlov

**Affiliations:** 1 Department of Bioengineering, Imperial College London, London, United Kingdom; 2 Laboratoire Physico-Chimie Curie, Institut Curie, PSL Research University, Sorbonne Université, CNRS, Paris, France

## Abstract

Hearing relies on the conversion of mechanical stimuli into electrical signals. In vertebrates, this process of mechanoelectrical transduction (MET) is performed by specialized receptors of the inner ear, the hair cells. Each hair cell is crowned by a hair bundle, a cluster of microvilli that pivot in response to sound vibrations, causing the opening and closing of mechanosensitive ion channels. Mechanical forces are projected onto the channels by molecular springs called tip links. Each tip link is thought to connect to a small number of MET channels that gate cooperatively and operate as a single transduction unit. Pushing the hair bundle in the excitatory direction opens the channels, after which they rapidly reclose in a process called fast adaptation. It has been experimentally observed that the hair cell’s biophysical properties mature gradually during postnatal development: the maximal transduction current increases, sensitivity sharpens, transduction occurs at smaller hair-bundle displacements, and adaptation becomes faster. Similar observations have been reported during tip-link regeneration after acoustic damage. Moreover, when measured at intermediate developmental stages, the kinetics of fast adaptation varies in a given cell, depending on the magnitude of the imposed displacement. The mechanisms underlying these seemingly disparate observations have so far remained elusive. Here, we show that these phenomena can all be explained by the progressive addition of MET channels of constant properties, which populate the hair bundle first as isolated entities and then progressively as clusters of more sensitive, cooperative MET channels. As the proposed mechanism relies on the difference in biophysical properties between isolated and clustered channels, this work highlights the importance of cooperative interactions between mechanosensitive ion channels for hearing.

## Significance

Hair cells are the sensory receptors of the inner ear that convert mechanical stimuli into electrical signals transmitted to the brain. Sensitivity to mechanical stimuli and the kinetics of mechanotransduction currents change during hair-cell development. The same trend, albeit on a shorter timescale, is also observed during hair-cell recovery from acoustic trauma. Furthermore, the current kinetics in a given hair cell depends on the stimulus magnitude, and the degree of that dependence varies with development. These phenomena have so far remained unexplained. Here, we show that they can all be reproduced using a single unifying mechanism: the progressive formation of clusters of channels, whose sensitivity is sharpened by cooperative gating.

## Introduction

Mechanoelectrical transduction (MET) is a fundamental process that transforms auditory stimuli into electrical signals that propagate to the brain. In vertebrates, this transformation takes place when a mechanical stimulus deflects the stereocilia of sensory receptors in the inner ear, the hair cells, opening mechanosensitive ion channels (1, 2). Stereocilia are actin-filled, enlarged microvilli arranged in a staircase manner in a hair bundle. Each stereocilium connects to its taller neighbor by a filamentous linkage, the tip link, necessary for mechanotransduction (3).

Tip links are elastic elements that tense or relax in response to the hair bundle’s deflections and transmit force onto the MET channels, changing their opening probability (4). When a hair bundle is deflected toward its tall edge, referred to as the positive direction, the MET channels open but then close again with time (5). This phenomenon, named adaptation, appears as a combination of two distinct processes: the first one is “fast,” with a time constant of a few milliseconds or even less in mammals, and the second one is “slow,” with a time constant of a few tens of milliseconds. Fast adaptation is accompanied by a rapid movement of the hair bundle in the direction opposite to the stimulus, called “the twitch” (6, 7). It is believed to be caused by the direct reclosure of the MET channels and is at least partially dependent on the action of Ca^2+^ ions (7, 8, 9, 10). Slow adaptation, in contrast, compels the hair bundle to move in the direction of the stimulus and affects the channels’ open probability via tip-link tension regulated by myosin motors (11).

Mechanotransduction in hair cells matures gradually before the onset of hearing (12, 13, 14). In mice and rats, cochlear hair cells are insensitive to mechanical stimuli before birth, and they become progressively functional along the cochlea’s tonotopic gradient; transduction currents appear first at birth or postnatal day 0 (P0) in hair cells at the base of the cochlea and from postnatal day 2 (P2) in those at the apex (13, 14). Four salient aspects of MET have been observed to evolve with postnatal development. First, the peak transduction current increases. Second, the interval of hair-bundle displacements over which channels gate decreases. Next, channels open typically at smaller displacements with respect to the resting position of the hair bundle. Finally, adaptation becomes faster and more complete (13, 14).

Apparently unrelated to these changes throughout postnatal development is the dependence of fast adaptation on other experimental conditions. When measured at intermediate developmental stages, the speed of fast adaptation in a given cell decreases as a function of the magnitude of the imposed displacement as can be seen from currents recorded in rat outer hair cells (Fig. 5 E in (14)) and in mouse inner hair cells (Fig. 3 C in (15)). In addition, hair cells with larger maximal MET currents display faster adaptation (Fig. 2 in (9)). These results are not limited to mammalian hair cells and are not restricted to postnatal development; similar observations have been made in turtle hair cells (16, 17) and in embryonic chicken hair cells (12), suggesting a generic property of MET.

Similar trends have been observed in hair cells recovering from the severing of their tip links, a common effect of exposure to loud sounds (15, 18, 19). Tip links regenerate, and mechanosensitivity is fully recovered after 48 h in mouse hair cells in culture (15, 19). During tip-link recovery over 2 days, the MET current undergoes changes that mirror those in developing hair cells over 7 days. At first, the cell shows no mechanotransduction. After 6 h, a small transduction current exists, but it displays little or no adaptation. As regeneration continues, the peak current increases, and adaptation appears and then becomes progressively faster and more complete (15).

These results are difficult to explain within the standard theoretical framework of MET—the classical gating-spring (GS) model—which is fundamentally a single-channel model (2, 4). It is unclear, in particular, how fast adaptation could evolve during postnatal development or during tip-link regeneration, given that it is likely an intrinsic property of MET channels (14, 20, 21). One hypothesis is that a change in hair-cell Ca^2+^ buffering capacity due to a temporal gradient of PMCA2 (Ca^2+^ pump) expression or perhaps a change in the subunit composition of the channel could lead to the observed changes of the kinetics (13, 22, 23). For example, outer hair cells differentially express two members of the transmembrane channel-like protein family during development (24, 25, 26). These proteins are believed to be pore-forming subunits of the MET channel and show different affinities to Ca^2+^, which could affect adaptation (22, 23). However, in apical hair cells in the mouse, transmembrane channel-like 1 proteins only begin to localize to stereocilia tips at postnatal day 6 (P6), whereas by that time, the kinetics of adaptation have already matured (27). Moreover, changes in a protein expression in general cannot explain the corresponding phenomena in hair cells recovering from a loss of tip links because tip-link regeneration and recovery of mechanotransduction do not require new protein synthesis (18). They also cannot explain why, at intermediate stages of development, adaptation is slower for larger imposed displacements (14, 15) and why it is faster for hair cells that display larger maximal currents (9).

Here, we propose a unifying explanation for the changes in the biophysical properties of the MET current during maturation of mechanotransduction, both in developing hair cells and during tip-link regeneration, as well as for the dependence of the kinetics of fast adaptation on the imposed hair-bundle displacement. We show that these phenomena can all be explained by the existence of two-channel populations with different transduction properties. First, in developing hair cells or one recovering after acoustic trauma, the relative proportion of these two populations varies with time, therefore changing the hair cell’s biophysical properties. Second, at a given time during development or tip-link regeneration, these populations are fixed but engage differently as a function of hair-bundle displacement, making the average adaptation kinetics position dependent.

## Model

### Theoretical description of postnatal MET maturation

In the experimental data obtained by Lelli et al. (13) from outer hair cells of the mouse cochlea, the number of functional MET channels increases during postnatal development as indicated both by the increased current amplitude and uptake of the fluorescent dye FM1-43. More precisely, there is no transduction current before birth, and, by about postnatal days 6 to 8, mechanotransduction reaches its adult characteristics. Similar observations have been made by Waguespack et al. (14) in outer hair cells of the rat cochlea from P0 to about postnatal day 7 (P7).

**Figure 1:**
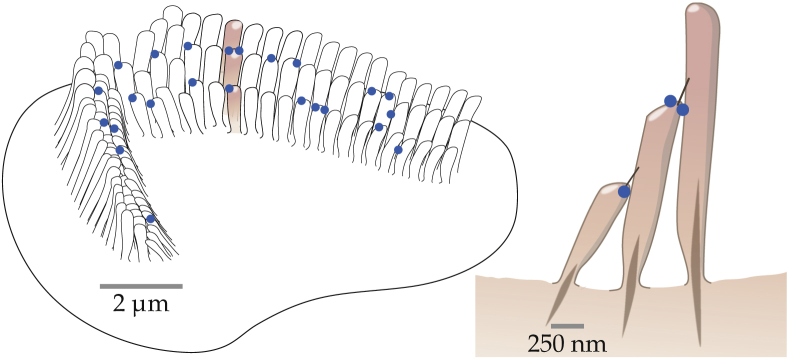
Left: schematic representation of the apical view of a hair bundle from an outer hair cell of a mammalian cochlea with a possible distribution of functional MET channels at an intermediate stage of development. Each blue dot represents a functional MET channel. Scale bar, ∼2 *μ*m. Right: cross-section representation of the series of three stereocilia shaded in the left panel. The two tip links connecting the adjacent stereocilia are represented as straight black lines, together with their associated functional channels as blue dots. Scale bar, ∼250 nm. To see this figure in color, go online.

It is not known precisely how the MET machinery is formed, nor is it known how many channels each tip link is connected to in the fully mature hair bundle. Here, in agreement with structural and functional data (28, 29) and as proposed in our recent model (30), we assume one channel per tip-link branch in mature hair cells, corresponding to two channels per tip link. We further hypothesize that, during the maturation process, individual MET channels connect to the tip links stochastically, populating the remaining available tip-link sites one by one with equal rates. As a result, at any time step during postnatal development, each tip link can find itself in one of the three following configurations: 1) the tip link is not connected to any channel, 2) the tip link is connected to a single channel as in the classical GS model (4), forming a “single-channel transduction unit,” or 3) the tip link is connected to two channels as in the model of (30), forming a “paired-channel transduction unit.” Following these rules, we illustrate in Fig. 1 a possible distribution of functional MET channels in a hair bundle at an intermediate stage of development.

### Population growth of MET channels

To distribute the channels across the hair bundle at any given time during postnatal development, one needs to specify how their number increases with the developmental stage of the cell. In the experiments of Lelli et al. (13) and Waguespack et al. (14), the peak MET current increases sigmoidally during development. Assuming a constant single-channel conductance, a realistic model is therefore a sigmoidal increase of the number of channels, as described by the following logistic function:

(1) $$n_{\text{ch}}\left(\text{P}\zeta \right)=\frac{n_{\text{max}}}{1+\exp\left[-\nu \left(\zeta -\bar{\zeta }\right)\right]},$$

where *n*_max_ is the total number of MET channels present in the mature hair cell, and *ν* and $\bar{\zeta }$ are fitting parameters. Fitting the curves reported in (14) with a maturation time of 7 days, we obtain $\bar{\zeta }=3.5$ days and *v* = 1.26 per day. For a hair bundle with 50 tip links (4) and a maximum of two channels per tip link, we further take *n*_max_ = 100 channels. The data of (13) for the mouse cochlea, however, point to a maturation time of 6 days. In that case, the same dependence applies with a simple rescaling of the phenomenological parameters, leading to $\bar{\zeta }=3.0$ days and *v* = 1.46 per day. In general, any reasonably smooth function that increases over the same period of time from zero or one to *n*_max_ would be valid here as well. To illustrate this point, we show in the Supporting Material results obtained with a linear increase of the number of channels, over the same period of time. In Table S1, we report the values given by Eq. 1 as well as by a linear increase with *n*_max_ = 100 and both total maturation times of 6 and 7 days, rounded up at each developmental stage to the closest integer number. In the following, we aim to compare our simulation results with the data of Lelli et al. (13) and Waguespack et al. (14). We therefore use the values reported here for the sigmoidal increase in the number of channels, with a maturation time of 6 and 7 days, respectively.

### The most likely number of channel pairs

**Figure 2:**
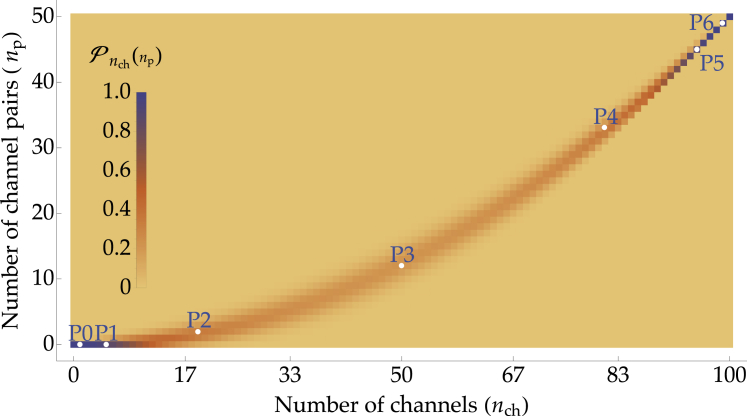
Heat map of the probability distribution of the number of channel pairs *n*_p_ for each value of the total number of channels *n*_ch_ as given by Eq. 3 in a hair bundle with 50 tip links (4). The white dots pinpoint the most likely numbers of channel pairs ${\bar{n}}_{\text{p}}$ across development from P0 to P6, assuming a sigmoidal growth of the number of channels *n*_ch_ as given by Eq. 1, with *n*_max_ = 100 channels, $\bar{\zeta }=3$ days, and *v* = 1.46 per day. To see this figure in color, go online.

To simulate how mechanotransduction changes with hair-cell development, we need to specify how these MET channels distribute across the hair bundle’s transduction sites at each developmental stage. For a hair bundle comprising *n*_t_ tip links connected to a total of *n*_ch_ channels, with a maximum of two channels per tip link, the distribution of channels is set by the number *n*_p_ of paired-channel units and the following constraints:

(2) $$\{\begin{array}{c} n_{\text{ch}}=2n_{\text{p}}+n_{\text{s}}\\ n_{\text{t}}=n_{\text{p}}+n_{\text{s}}+n_{\text{e}} \end{array}\;,$$

where *n*_s_ is the number of single-channel units and *n*_e_ that of tip links connecting to zero channels. Assuming that the channels are added one by one with equal probability to any of the remaining empty sites, the probability of having *n*_p_ formed pairs in the hair bundle reads as follows:

(3) $$P_{n_{\text{ch}}}\left(n_{\text{p}}\right)=\frac{\left(\begin{array}{c} n_{\text{t}}\\ n_{\text{p}} \end{array}\right)\cdot 2^{n_{\text{ch}}-2n_{\text{p}}}\;\left(\begin{array}{c} n_{\text{t}}-n_{\text{p}}\\ n_{\text{ch}}-2n_{\text{p}} \end{array}\right)}{\left(\begin{array}{c} 2n_{\text{t}}\\ n_{\text{ch}} \end{array}\right)}\;,$$

where the parentheses represent the binomial distribution (31). This equation states that once the *n*_p_ pairs have been distributed across the $n_{\text{t}}$ tip links, *n*_ch_ − 2*n*_p_ single channels remain to be distributed across the *n*_t_ − *n*_p_ available tip links, with two choices per channel. The denominator corresponds to the total number of possible distributions of *n*_ch_ channels in 2*n*_t_ locations. This probability distribution is represented in Fig. 2 as a heat map for each value of the total number of channels *n*_ch_ between 0 and 100.

In the following, we assume that the distribution of channels in the hair bundle corresponds to the configuration of maximal likelihood derived from Eq. 3. The white dots in Fig. 2 indicate the most likely state at each postnatal day from P0 to P6, assuming a sigmoidal growth of the number of channels in a hair bundle with 50 tip links that develops in 6 days. In Table S1, we report the values of the most likely number of channel pairs ${\bar{n}}_{\text{p}}$, rounded up to the closest integer for every postnatal day from P0 to maturation (P6/P7), for both the linear and sigmoidal growths of the number of channels.

### Open probability curves

Having determined the most likely number of channel pairs ${\bar{n}}_{\text{p}}$ as a function of the developmental stage P*ζ*, we now study how the biophysical properties of the MET current change with development. In particular, we investigate the evolution of the open probability as a function of displacement at the level of the entire hair bundle and compare our model results with the experimental data of (13, 14).

The total response current of a hair cell is the sum of the currents through all open MET channels, all of which are assumed to carry the same individual channel current. Single and paired units are expected to respond differently to hair-bundle displacements and are therefore described by different open probability curves. This is because the open paired channels have an energetically favorable interaction through the lipid bilayer, a key contribution to the total energy balance of the system, which isolated channels lack. Therefore, channels in pairs open more readily than isolated ones. It follows that whenever the ratio of paired to single channels changes, the open probability curve for the whole hair bundle changes accordingly.

To estimate the resulting open probability curve, we rely on the finding that tip links within a hair bundle are mechanically coupled in parallel (32, 33, 34, 35, 36). We assume the same geometrical projection factor *γ* between the oblique orientation of each tip link and the axis of hair-bundle displacement, independently of the number of channels connected to that tip link (4). As a result, every unit is subjected to the same change of tip-link extension as a function of the hair-bundle displacement *X*. Therefore, the open probability curve for the entire hair bundle at the developmental stage P*ζ* corresponds to the average contribution over all units present in the hair bundle. It reads as follows:

(4) $$P_{\text{P}\zeta }^{\text{HB}}\left(X\right)=\frac{{\bar{n}}_{\text{s}}\left(\text{P}\zeta \right)\cdot P_{\text{s}}\left(X\right)+2\;{\bar{n}}_{\text{p}}\left(\text{P}\zeta \right)\cdot P_{\text{p}}\left(X\right)}{{\bar{n}}_{\text{s}}\left(\text{P}\zeta \right)+2\;{\bar{n}}_{\text{p}}\left(\text{P}\zeta \right)}\;,$$

where $P_{\text{s}}\left(X\right)$ is the open probability function of a single-channel unit, $P_{\text{p}}\left(X\right)$ is that of a paired-channel unit, and ${\bar{n}}_{\text{s}}\left(\text{P}\zeta \right)$ and ${\bar{n}}_{\text{p}}\left(\text{P}\zeta \right)$ correspond to the most likely numbers of single- and paired-channel units at the developmental stage P*ζ*, respectively. The factor 2 accounts for the difference in channel numbers between single and paired units and therefore for the difference in the contribution per unit to the total MET current, which is the experimentally measured quantity used to determine the open probability. We have discussed above how to determine ${\bar{n}}_{\text{s}}\left(\text{P}\zeta \right)$ and ${\bar{n}}_{\text{p}}\left(\text{P}\zeta \right)$. In the following paragraph, we specify our choice for $P_{\text{s}}\left(X\right)$ and $P_{\text{p}}\left(X\right)$.

We model the open probability curve for isolated channels $P_{\text{s}}\left(X\right)$ using the established GS model (4). Its analytic expression reads as follows:

(5) $$P_{\text{s}}\left(X\right)=\frac{1}{1+\text{exp}\left[-z\cdot \left(X-X_{1/2}\right)/\left(k_{\text{B}}T\right)\right]}\;,$$

where *k*_B_*T* is the thermal energy at temperature *T*, *z* is the gating force, and *X*_1/2_ is the position along the *X* axis for which half of the single channels are open (4).

The open probability curve $P_{\text{p}}\left(X\right)$ is derived from the paired-channel model introduced earlier, fully described in our previous work (30). In contrast to $P_{\text{s}}\left(X\right)$, no explicit analytic formula can be reported here as this function derives from a numerical procedure described in (30). Briefly, this model proposes that every tip link in a mature hair bundle is connected to two channels, as suggested by experiments (28, 37). Membrane deformations after the opening and closing of either of the two channels couple their open probabilities, and the two channels gate cooperatively. This model reproduces the physiological behavior of the hair bundle without invoking an unrealistically large conformation change of the channel upon gating—the gating swing—as required when fitting with the classical GS model (4, 10, 38, 39). Instead, the large change in tip-link extension upon channel gating results here from the relative movement of the paired channels in the membrane. Because it includes membrane-mediated interactions between the paired channels, this model is also in agreement with the observation that the lipid bilayer plays a role in modulating the channels’ gating properties as well as in tuning fast and slow adaptation (10, 40).

This model depends on a list of parameters specified in Table S2. Among these parameters, a first category encompasses those describing the structural and mechanical properties of the hair bundle. They are the tip-link stiffness *k*_t_, the gating swing *δ*, the projection factor *γ*, the difference in the energy of one channel between its open and closed conformations (or gating energy) *E*_g_, the total number of tip links *N*, and the total stiffness of the ensemble of stereociliary pivots *K*_sp_ (4). These parameters appear in the model for single-channel units as well, in which the gating force reads *z* = *γk*_t_*δ*. In addition, we must define parameters specific to the two-channel model, which describes channels that are mobile within the membrane and interacting via its deformations. Among these parameters are the length *l* of the branches of the tip-link fork, the stiffness *k*_a_ of the adaptation springs that anchor the channels to the actin network inside stereocilia, and a set of parameters describing the energy contribution of the stereociliary membrane, which depend on the state of the channel pair: open-open (OO), open-closed, or closed-closed (CC). We refer the reader to (30) for a full presentation.

**Figure 3:**
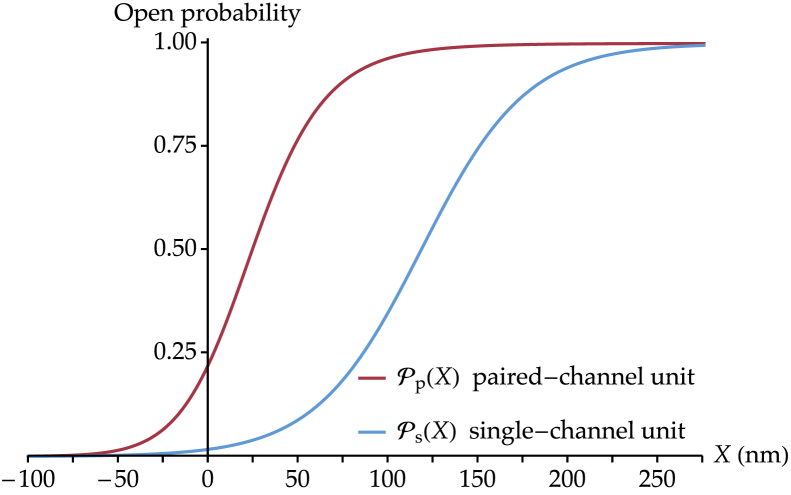
Curves of open probability versus hair-bundle displacement for the two types of units present in a developing hair bundle: paired-channel transduction units ($P_{\text{p}}\left(X\right)$, *red curve*) and single-channel transduction units ($P_{\text{s}}\left(X\right)$, *blue curve*). The parameters that are shared by the two functions take the following values: *γ* = 0.1, *k*_t_ = 0.7 mN⋅m^−1^ (41), *δ* = 2 nm (30), and *T* = 298 K. The parameters specific to the paired-channel model are set by our previous work (30) and are specified in Table S2. The origin of the *X* axis is such that the hair bundle is at rest at *X* = 0 in both models. This implies, in particular, *X*_1/2_ = 118 nm in Eq. 5 (see the Supporting Material). To see this figure in color, go online.

The resulting open probability curves are shown in Fig. 3, using the parameter values of Table S2. These values have been chosen to correspond to those measured in a recent biophysical study of mammalian cochlear hair cells (41), in which all the parameters relevant to this work have been estimated from the same data set. The open probability curve $P_{\text{p}}\left(X\right)$ is plotted in red, with the origin of the *X* axis set such that the hair bundle sits at *X* = 0 when no external force is applied. The open probability curve $P_{\text{s}}\left(X\right)$ is plotted in blue with the same parameter values, with *X*_1/2_ set such that the hair bundle sits at *X* = 0 when no external force is applied, similarly to the paired-channel model above. We can see that, although the parameters common to both models have identical values, the open probability curve from the two-channel model describes channels that gate at smaller displacements and is steeper than that from the classical GS model, corresponding to a greater sensitivity. These results are in agreement with studies performed on pairs of mechanosensitive channels of large conductance in *Escherichia coli*, which have shown that closely apposed channels open at a smaller membrane tension than isolated ones and display steeper open probability curves, again corresponding to a higher sensitivity (42).

## Results

### Leftward shift of the open probability curve

**Figure 4:**
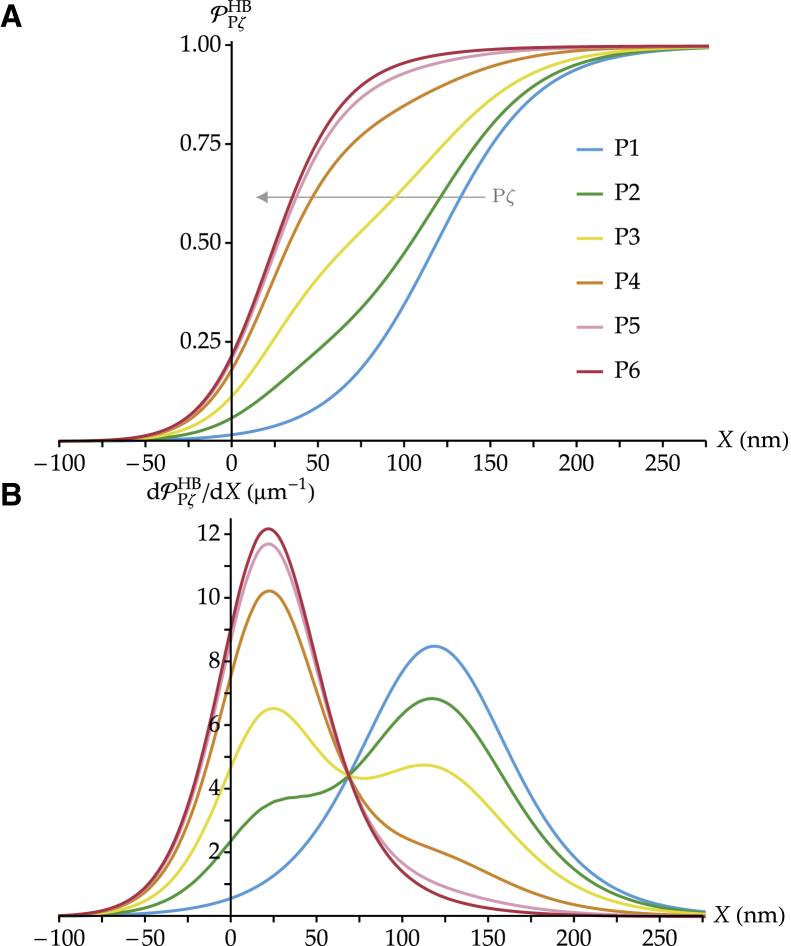
(*A*) Simulated maturation of the open probability versus displacement curve $P_{\text{P}\zeta }^{\text{HB}}\left(X\right)$ in a developing hair cell. We report the case of a sigmoidal growth of the number of MET channels over 6 days, as given in Table S1. The parameters that define the transduction units are the same as in Fig. 3. The gray arrow indicates the progression toward increasing values of the parameter P*ζ* across the curves. (*B*) Derivatives of the open probability curves of (*A*), using the same color code. The open probability curve shifts leftward with development; the hair cell becomes progressively more sensitive, and its working range decreases accordingly. To see this figure in color, go online.

The open probability curve recorded from hair cells of the rat cochlea shifts toward smaller displacements during postnatal development (14). To compare our model results with these measurements, we plot in Fig. 4 *A* the simulated open probability versus displacement curves for the entire hair bundle across development as given by Eq. 4 in the case of a sigmoidal increase of the number of channels over 6 days (see Table S1). In Fig. 4 *B*, we plot the derivatives of these open probability curves to represent the hair-bundle sensitivity. A maximum of sensitivity corresponds to a maximum of the open probability derivative, for which a maximal number of channels gate per unit hair-bundle displacement. At early developmental stages, most tip links connect to zero or one channel, and the open probability curve resembles that of a single-channel unit (postnatal day 1 (P1) curve here, to be compared with the $P_{\text{s}}$ curve of Fig. 3). The sensitivity peaks for a displacement *X* around 125 nm at ∼8.5 *μ*m^−1^, which corresponds to the maximal sensitivity of single channels. At late developmental stages, most tip links are connected to two channels, and the open probability curves resemble that of a paired-channel unit (P6 curve here, to be compared with the $P_{\text{p}}$ curve of Fig. 3). The sensitivity peaks for a displacement *X* around 25 nm where paired channels gate and is roughly 50% higher than that of single channels. At intermediate developmental stages, the open probability curve appears as a weighted average of these two extremes and evolves with development by shifting toward smaller hair-bundle displacements as the number of single-channel units decreases and that of paired-channel units increases. Analogous results can be observed in the case of a linear growth in the number of channels. In Fig. S1, we report the evolution of the open probability curve across development (Fig. S1 A) and its derivative (Fig. S1 B) in a hair bundle reaching maturation linearly in 6 days (see Table S1).

Because of the presence of two different MET units that gate typically at different hair-bundle displacements and with two different sensitivities, the open probability versus displacement curves at intermediate stages of development present an asymmetric shape; compared to a standard sigmoid, they increase more rapidly at small displacements and then rise more gently to saturation, which resembles the shapes of the corresponding curves obtained experimentally (13). This phenomenon is comparable to what has been observed in voltage-dependent Ca^2+^ channels. In that case, data suggest the existence of two populations of ion channels as well, usually called “willing” and “reluctant” (43). The asymmetric shape of the open probability curves is due to the fact that these two channel populations open at different voltages, similarly to what we propose here in terms of hair-bundle displacements.

### Decrease of the operating range

**Figure 5:**
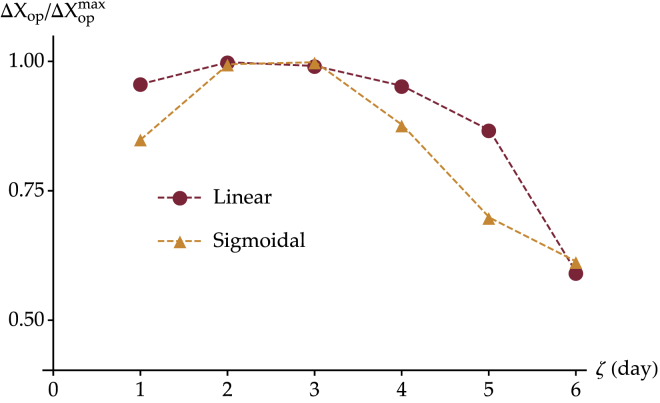
Simulated operating range normalized with respect to its maximal value and plotted between 0.50 and 1.00. Both cases of linear (*red circles*) and sigmoidal (*orange triangles*) growth functions of the number of channels over 6 days are reported. To see this figure in color, go online.

As outer hair cells mature, their operating range decreases and stabilizes by P6/P7 at a value that is roughly 50% that recorded at P0, both in rat and mouse cochleas (13, 14). This narrowing of the operating range is linked to an increase of the slope of the open probability versus displacement curve, reflecting an increased sensitivity of the hair cell to mechanical stimuli. To compare our simulation with these experimental data quantitatively, we choose as the operating range *ΔX*_op_ the difference in hair-bundle displacements corresponding respectively to 95 and 5% of the hair-bundle open probability: $\Delta X_{\text{op}}=X_{P^{\text{HB}}=0.95}-X_{P^{\text{HB}}=0.05}$. In Fig. 5, we plot this quantity at different developmental stages normalized by its maximal value. For a comparison, we report these results for both the linear and sigmoidal increases of the number of channels over 6 days (see Table S1).

### Maturation of adaptation

The fast and slow components of adaptation at the level of an entire hair bundle are characterized by two phenomenological time constants, $\tau_{\text{fast}}^{\text{exp}}$ and $\tau_{\text{slow}}^{\text{exp}}$. They are obtained by fitting the experimentally measured response current *I*(*t*) of the hair cell as a function of the time *t* with the following expression:

(6) $$I\left(t\right)=I_{A}+I_{B}\;\text{exp}\left(-\frac{t}{\tau_{\text{fast}}^{\text{exp}}}\right)+I_{C}\;\text{exp}\left(-\frac{t}{\tau_{\text{slow}}^{\text{exp}}}\right)\;.$$

Here, *I*_*A*_ is a baseline current, and *I*_*B*_ and *I*_*C*_ determine the contributions of fast and slow adaptation, respectively. Typical values of $\tau_{\text{fast}}^{\text{exp}}$ in mammalian hair cells range from 0.05 to 0.6 ms, whereas $\tau_{\text{slow}}^{\text{exp}}$ ranges from 4 to 16 ms (8, 9, 13, 14).

In rat and mouse hair cells, these two phenomenological time constants decrease during early postnatal development (13, 14). In some cases, outer hair cells initially display mechanosensitivity but no form of adaptation (14, 44). Here, we propose that these results betray the existence of the two channel populations introduced above, which, in addition to having different open probability versus displacement curves, also have different adaptation kinetics. Similarly as before, we propose that it is the change in their relative proportions that leads to the observed changes in the phenomenological time constants of fast and slow adaptation at the level of the entire hair bundle, even though each individual channel population has adaptation time constants that are fixed across postnatal development.

Taking into account the existence of the two channel populations, the response current *I*_P*ζ*,*X*_(*t*) of the hair cell at a developmental stage P*ζ* and for an imposed step displacement *X* can be written as follows:

(7) $$\begin{array}{c} I_{\text{P}\zeta ,X}\left(t\right)={\bar{n}}_{\text{s}}\left(\text{P}\zeta \right)\;P_{\text{s}}\left(X\right)\;i_{\text{ch}}\\ \times \left\{a^{\text{s}}+b^{\text{s}}\;\text{exp}\left(-\frac{t}{\tau_{\text{fast}}^{\text{s}}}\right)+c^{\text{s}}\;\text{exp}\left(-\frac{t}{\tau_{\text{slow}}^{\text{s}}}\right)\right\}\\ +2{\bar{n}}_{\text{p}}\left(\text{P}\zeta \right)\;P_{\text{p}}\left(X\right)\;i_{\text{ch}}\\ \times \left\{a^{\text{p}}+b^{\text{p}}\;\text{exp}\left(-\frac{t}{\tau_{\text{fast}}^{\text{p}}}\right)+c^{\text{p}}\;\text{exp}\left(-\frac{t}{\tau_{\text{slow}}^{\text{p}}}\right)\right\}\;. \end{array}$$

Here, ${\bar{n}}_{\text{s}}\left(\text{P}\zeta \right)$ and ${\bar{n}}_{\text{p}}\left(\text{P}\zeta \right)$ indicate the most likely numbers of single- and paired-channel units at P*ζ*, as discussed previously and reported in Table S1. The parameter *i*_ch_ is the current passing through one open MET channel, whereas $P_{\text{s}}\left(X\right)$ and $P_{\text{p}}\left(X\right)$ are the open probabilities of single and paired channels, respectively, at the imposed displacement *X* and before adaptation takes place (see Open probability curves, Eq. 5, and the accompanying text). Together, the product ${\bar{n}}_{\text{s}}\left(\text{P}\zeta \right)\cdot P_{\text{s}}\left(X\right)\cdot i_{\text{ch}}$ (respectively $2{\bar{n}}_{\text{p}}\left(\text{P}\zeta \right)\cdot P_{\text{p}}\left(X\right)\cdot i_{\text{ch}}$) represents the total current passing through single channels (respectively paired channels) in the hair bundle before adaptation takes place. The dimensionless variables *b*^s(p)^ and *c*^s(p)^ weigh the relative contributions of the fast and slow components of adaptation for the single (s) or paired (p) channels, and *a*^s(p)^ = 1 − *b*^s(p)^ − *c*^s(p)^ gives the relative amplitude of the remaining currents after adaptation has taken place. The four time constants $\tau_{\text{fast}}^{\text{s}\left(\text{p}\right)}$ and $\tau_{\text{slow}}^{\text{s}\left(\text{p}\right)}$ represent the characteristic times over which the channels undergo fast and slow adaptation, respectively, with specific values for single and paired channels. This equation states that the total decline in response current is the weighted sum of the contributions of the two types of MET units, each adapting at its own pace by the two independent processes of fast and slow adaptation.

To reduce the number of parameters, we assume that the amplitudes of fast and slow adaptation are the same with *b*^s(p)^ = *c*^s(p)^. We then obtain the following:

(8) $$\begin{array}{c} I_{\text{P}\zeta ,X}\left(t\right)={\bar{n}}_{\text{s}}\left(\text{P}\zeta \right)\;P_{\text{s}}\left(X\right)\;i_{\text{ch}}\\ \times \left\{a^{\text{s}}+\frac{1-a^{\text{s}}}{2}\left[\text{exp}\left(-\frac{t}{\tau_{\text{fast}}^{\text{s}}}\right)+\text{exp}\left(-\frac{t}{\tau_{\text{slow}}^{\text{s}}}\right)\right]\right\}\\ +2{\bar{n}}_{\text{p}}\left(\text{P}\zeta \right)\;P_{\text{p}}\left(X\right)\;i_{\text{ch}}\\ \times \left\{a^{\text{p}}+\frac{1-a^{\text{p}}}{2}\left[\text{exp}\left(-\frac{t}{\tau_{\text{fast}}^{\text{p}}}\right)+\text{exp}\left(-\frac{t}{\tau_{\text{slow}}^{\text{p}}}\right)\right]\right\}\;, \end{array}$$

leaving four time constants ($\tau_{\text{fast}}^{\text{s}\left(\text{p}\right)}$ and $\tau_{\text{slow}}^{\text{s}\left(\text{p}\right)}$) and two relative amplitudes (*a*^s(p)^) as fitting parameters of the entire model for the normalized current. In the experimental data of (13, 14), adaptive currents are measured by imposing step deflections onto the hair bundle such that the ensemble open probability is 50% at the onset of adaptation. To reproduce these results, we investigate the adaptation current for an imposed step displacement *X*_HB,50%_, at which the hair-bundle open probability $P_{\text{P}\zeta }^{\text{HB}}$ as given by Eq. 4 equals 50%, for each developmental stage P*ζ*. We set the parameters entering Eq. 8 by using the experimentally measured amplitudes of the MET currents before and after adaptation, both at early and late stages of hair-cell development. At P1, according to our model, most functional MET units contain only one channel (13, 14) (see Table S1 for the sigmoidal increase of the number of channels). Therefore, the hair-bundle currents at the onset of adaptation and after adaptation has taken place can be written, respectively:

(9) $$\begin{array}{l} I_{\text{P}1}\left(t=0\right)≃\frac{1}{2}n_{\text{ch}}\left(\text{P}1\right)\;i_{\text{ch}}\\ I_{\text{P}1}\left(t\gg \tau_{\text{slow}}^{\text{s}}\right)≃\frac{1}{2}n_{\text{ch}}\left(\text{P}1\right)\;i_{\text{ch}}\;a^{\text{s}}\;. \end{array}$$

These expressions allow us to express the extent of adaptation, defined at the developmental stage P*ζ* as $1-I_{\text{P}\zeta }\left(t\gg \tau_{\text{slow}}^{\text{s},\text{p}}\right)/I_{\text{P}\zeta }\left(t=0\right)$ (13, 14). At P1, this quantity is simply (1 − *a*^s^). For the same reason, the time constants $\tau_{\text{fast}}^{\text{s}}$ and $\tau_{\text{slow}}^{\text{s}}$ are estimated directly from the time traces of the currents recorded at P1. The same reasoning leads to the corresponding quantities at the end of maturation when all channels are paired at P6 or P7, depending on which data set (13, 14) we consider:

(10) $$\begin{array}{l} I_{\text{P}6/7}\left(t=0\right)=\frac{1}{2}n_{\text{ch}}\left(\text{P}6/7\right)\;i_{\text{ch}}\\ I_{\text{P}6/7}\left(t\gg \tau_{\text{slow}}^{\text{p}}\right)=\frac{1}{2}n_{\text{ch}}\left(\text{P}6/7\right)\;i_{\text{ch}}\;a^{\text{p}}\;. \end{array}$$

Similarly, the extent of adaptation at P6/7 is (1 − *a*^p^), and the time constants $\tau_{\text{fast}}^{\text{p}}$ and $\tau_{\text{slow}}^{\text{p}}$ are estimated from the time traces recorded at that developmental stage.

**Figure 6:**
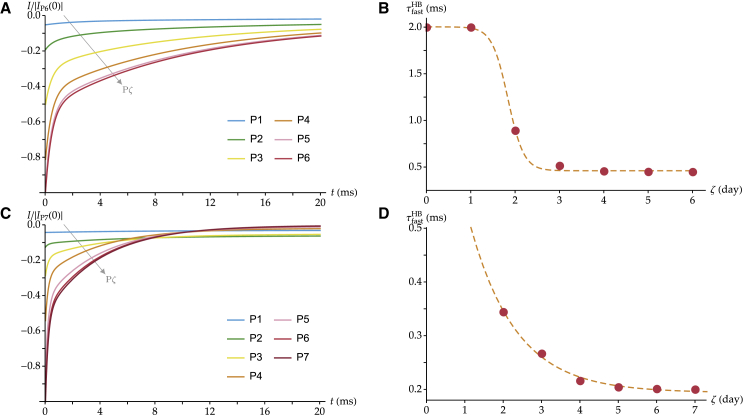
Normalized response currents (*A* and *C*) and time constants of fast adaptation for the entire hair bundle (*B* and *D*) across simulated postnatal development. (*A*) The response currents are generated using Eq. 8 with a sigmoidal growth of the number of channels over 6 days (see Table S1) and the following parameter values: *a*^s^ = 0.20, $\tau_{\text{fast}}^{\text{s}}=2$ ms, $\tau_{\text{slow}}^{\text{s}}=20$ ms, *a*^p^ = 0.05, $\tau_{\text{fast}}^{\text{p}}=0.45$ ms, and $\tau_{\text{slow}}^{\text{p}}=10$ ms. For each curve, *X* is computed such that $P_{\text{P}\zeta }^{\text{HB}}\left(X\right)$ is equal to 0.5, as given by Eq. 4. The gray arrow indicates the progression toward increasing values of the parameter P*ζ* across the different curves. (*B*) The time constant of fast adaptation at the level of the entire hair bundle $\tau_{\text{fast}}^{\text{HB}}$ is reported as a function of the postnatal day P*ζ*, obtained by fitting the curves of (*A*) with Eq. 6. These results aim at reproducing the data in Lelli et al. from the mouse cochlea (13). (*C*) and (*D*) A similar procedure is followed to reproduce the data in Waguespack et al. from the rat cochlea (14). (*C*) The response currents are generated using Eq. 11 with a sigmoidal growth of the number of channels over 7 days (see Table S1) and the following parameter values: *a*^s^ = 0.7, $\tau_{\text{slow}}^{\text{s}}=8$ ms, $a^{\text{p}}=0$, $\tau_{\text{fast}}^{\text{p}}=0.2$ ms, and $\tau_{\text{slow}}^{\text{p}}=4$ ms. In (*B* and *D*), the results are displayed together with their best fits to a sigmoidal and an exponential function, respectively, as done in (13, 14) (*dashed lines*). To see this figure in color, go online.

In the basal region of the mouse cochlea, the extent of adaptation ranges from 80% at P0 to 95% at P6 (13), setting *a*^s^ = 0.20 and *a*^p^ = 0.05. In addition, we obtain $\tau_{\text{fast}}^{\text{s}}=2$ ms and $\tau_{\text{slow}}^{\text{s}}=20$ ms as the adaptation time constants measured at P1 and $\tau_{\text{fast}}^{\text{p}}=0.45$ ms and $\tau_{\text{slow}}^{\text{p}}=10$ ms at P6 (13). We display in Fig. 6 *A* the resulting simulated currents at the level of the entire hair bundle as given by Eq. 8 from P1 to P6. Each current is normalized with respect to its value with half of the channels open in the fully developed hair cell *I*_P6_(0) = (*n*_ch_(P6)/2) × *i*_ch_, corresponding to the maximal current of the hair cell at the imposed displacement *X*_HB,50%_. We can see an inflection in the response-current curves after postnatal day 3, when the double exponential becomes clearly visible, corresponding to two distinct kinetics of the overall fast and slow adaptation components at the level of the entire hair bundle.

The simulated curves display a large variation in their apparent relaxation times. To quantify this variability, we fit these curves with a double exponential as in Eq. 6 as if the simulated curves were experimental data. We label the obtained adaptation time constants at the level of the entire hair bundle $\tau_{\text{fast}}^{\text{HB}}$ and $\tau_{\text{slow}}^{\text{HB}}$ to distinguish them from their experimentally derived counterparts $\tau_{\text{fast}}^{\text{exp}}$ and $\tau_{\text{slow}}^{\text{exp}}$. We report in Fig. 6 *B* the values of $\tau_{\text{fast}}^{\text{HB}}$ obtained across development from P1 to P6. In addition, at P0, we have a single isolated channel in the simulated hair bundle, such that $\tau_{\text{fast}}^{\text{HB}}=\tau_{\text{fast}}^{\text{s}}$. We can see that $\tau_{\text{fast}}^{\text{HB}}$ decreases with development, in agreement with the experimental findings (13). A similar global trend holds true for slow adaptation (see Fig. S2 *A*). Overall, these results are in agreement with the experimental observations of (13).

The same procedure can be applied to simulate adaptation as observed in the rat cochlea (14). Some of these experiments show an absence of fast adaptation at early stages of development. Within our framework, this suggests that isolated channels might only be capable of slow adaptation in that case. In contrast, the paired channels would still display both components of adaptation, as observed in the fully developed hair cells. Note that this hypothesis does not preclude the possibility that some hair cells already display a fast component of adaptation at birth, in the case in which some paired channels are already present at that developmental stage. Under these hypotheses, Eq. 8 is replaced by:

(11) $$\begin{array}{c} I_{\text{P}\zeta ,X}\left(t\right)={\bar{n}}_{\text{s}}\left(\text{P}\zeta \right)\;P_{\text{s}}\left(X\right)\;i_{\text{ch}}\\ \times \left\{a^{\text{s}}+\left(1-a^{\text{s}}\right)\left[\text{exp}\left(-\frac{t}{\tau_{\text{slow}}^{\text{s}}}\right)\right]\right\}\\ +2{\bar{n}}_{\text{p}}\left(\text{P}\zeta \right)\;P_{\text{p}}\left(X\right)\;i_{\text{ch}}\\ \times \left\{a^{\text{p}}+\frac{1-a^{\text{p}}}{2}\left[\text{exp}\left(-\frac{t}{\tau_{\text{fast}}^{\text{p}}}\right)+\text{exp}\left(-\frac{t}{\tau_{\text{slow}}^{\text{p}}}\right)\right]\right\}\;. \end{array}$$

Experimentally, the characteristics of adaptation were only measured in the basal part of the rat cochlea for the cells that displayed fast adaptation at P1. Assuming that the extent of adaptation as well as the slow adaptation time constant are the same in all basal cells at that developmental stage, we can use these measurements to simulate the cells that do not display fast adaptation at P1. These measurements indicate that the extent of adaptation is around 30% at P1 and reaches 100% by postnatal day 3/postnatal day 4 (P4) (14). This sets *a*^s^ = 0.7 and *a*^p^ = 0. In addition, the fitted kinetics of fast and slow adaptation at P1 and P7 give $\tau_{\text{slow}}^{\text{s}}=8$ ms, $\tau_{\text{fast}}^{\text{p}}=0.2$ ms, and $\tau_{\text{slow}}^{\text{p}}=4$ ms.

With these parameter values, we display in Fig. 6 *C* the normalized adaptation currents at different developmental stages P*ζ*. In Fig. 6 *D*, we report the corresponding fitted values of $\tau_{\text{fast}}^{\text{HB}}$ at each developmental stage, from P2 to P7. At P1, there are no paired-channel units in the model, and fast adaptation is absent. At later developmental stages, $\tau_{\text{fast}}^{\text{HB}}$ is finite. It then decreases with development and reaches ∼0.2 ms at maturation. These results are in agreement with the trend reported in (14, 44), in which adaptation has only been observed a few days after the appearance of mechanotransduction. Similarly to Fig. S2 *A*, we report in Fig. S2 *B* the corresponding decrease in $\tau_{\text{slow}}^{\text{HB}}$ as a function of P*ζ*.

**Figure 7:**
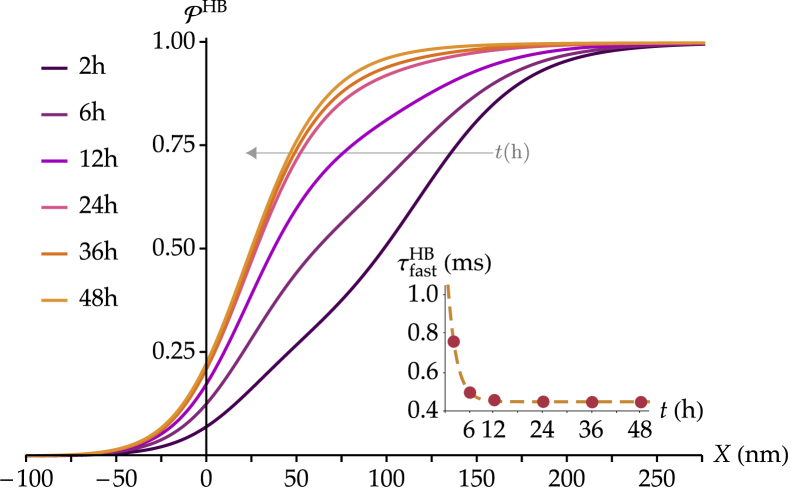
Evolution of the ensemble open-probability function in a simulated hair cell with 50 broken tip links after 2, 6, 12, 24, 36, and 48 h of recovery and corresponding evolution of $\tau_{\text{fast}}^{\text{HB}}$ (*inset*). The curves are derived from Eqs. 4 and 8 with the number of channels from Eq. 13 and the following parameters: *a*^s^ = 0.20, $\tau_{\text{fast}}^{\text{s}}=2$ ms, $\tau_{\text{slow}}^{\text{s}}=20$ ms, *a*^p^ = 0.05, $\tau_{\text{fast}}^{\text{p}}=0.45$ ms, and $\tau_{\text{slow}}^{\text{p}}=10$ ms. The factors $P_{\text{s}}\left(X\right)$ and $P_{\text{p}}\left(X\right)$ are calculated with an imposed displacement *X*_HB,50%_ such that $P_{\text{P}\zeta }^{\text{HB}}$ equals 50% before adaptation. The gray arrow indicates the progression toward increasing values of *t*—the duration of recovery—across the different curves. To see this figure in color, go online.

Data from Lelli et al. (13) suggest that MET channels in mouse hair cells display fast adaptation at P1, whereas Marcotti et al. observed hair cells incapable of adaptation at P2 in the same animal model (Fig. 7 in (44)). Waguespack et al. observed no fast adaptation in a fraction of rat hair cells at early postnatal developmental stages (14). In our analysis, we used single channels with or without fast adaptation to reproduce those experimental data, and we showed that the model accommodates either type of behavior. These apparent discrepancies within recorded data can be resolved assuming that fast adaptation at early stages of postnatal development results from paired-channel units already present in the hair bundle. At P1, rat basal hair cells in (14) display a peak current of ∼100 pA at a holding potential of −80 mV. This is approximately one third of that recorded in mice of the same age at a holding potential of −64 mV (13), an observation that is compatible with the possibility that the mouse hair cells in (13) possessed a greater number of paired channels at P1. Small currents (∼150 pA at −84 mV) have also been recorded at P2 in mouse preparations by Marcotti et al. (44), in which hair cells displayed no adaptation.

In agreement with this interpretation, we show in Fig. S3 that a similar trend in the maturation of the time constant of fast adaptation as the one observed in (13) can be generated, assuming that single-channel units are not capable of fast adaptation but that MET development starts before birth with paired channels already present at P1. In this figure, we represent $\tau_{\text{fast}}^{\text{HB}}$ as a function of P*ζ* using the same parameter values as in Fig. 6 *D* but with a channel population growth over a new total of 10 days and starting 3 days before birth to end at P7 as before. The hair cell displays a finite value of $\tau_{\text{fast}}^{\text{HB}}$ already at P0, as in (13) and our earlier Fig. 6 *B*, despite the fact that single-channel units, in this case, do not exhibit fast adaptation.

Our model also explains why it is possible to observe fast adaptation in some hair cells but not in others obtained from the same animal. At early developmental stages, the number of channel pairs *n*_p_ can vary from cell to cell, the actual values being taken from the probability distribution given by Eq. 3 and illustrated in Fig. 2. This means that, at early developmental stages, some hair bundles can contain paired-channel units, therefore displaying a certain amount of fast adaptation, whereas others do not.

### Tip-link regeneration

One of the effects of loud sounds on hair cells is the disruption of tip links. This effect is temporary, however, because tip links can regenerate (15, 18, 19, 45). In mouse hair cells in culture, this process takes ∼48 h (15), during which the biophysical changes of the MET current recapitulate those observed in postnatal development across 7 days; the peak current becomes larger, adaptation becomes faster and more complete, and the slope of the open probability curve increases (15, 18, 19).

To restore hair-cell mechanosensitivity, new tip links must establish connections with the MET channels. We simulate this process by assuming that each of the tip link’s two branches has a given probability per unit time $p_{\text{tip}}^{\text{s}}$ to connect to a MET channel and a probability per unit time $p_{\text{tip}}^{\text{p}}$ that the second branch connects to another MET channel, given that the first branch has already been linked. The evolution of the number of paired- and single-channel units is determined by the following equations:

(12) $$\{\begin{array}{c} {\dot{n}}_{\text{e}}=-p_{\text{tip}}^{\text{s}}n_{\text{e}}\\ {\dot{n}}_{\text{s}}=p_{\text{tip}}^{\text{s}}n_{\text{e}}-p_{\text{tip}}^{\text{p}}n_{\text{s}}\\ {\dot{n}}_{\text{p}}=p_{\text{tip}}^{\text{p}}n_{\text{s}} \end{array}\;,$$

where the variables *n*_e_, *n*_s_, and *n*_p_ have been defined previously (see Eq. 2 and accompanying text). Here, $\dot{n}$_e,s,p_ represent the time derivatives d*n*_e,s,p_/d*t*, respectively, where *t* is the time passed after tip-link damage on the order of several hours. For simplicity, we consider the case $p_{\text{tip}}^{\text{s}}=p_{\text{tip}}^{\text{p}}=p_{\text{tip}}$. Eq. 12 is then solved by the following:

(13) $$\{\begin{array}{c} n_{\text{e}}\left(t\right)=n\;e^{-p_{\text{tip}}t}\\ n_{\text{s}}\left(t\right)=np_{\text{tip}}t\;e^{-p_{\text{tip}}t}\\ n_{\text{p}}\left(t\right)=n\left[1-\left(1+p_{\text{tip}}t\right)\;e^{-p_{\text{tip}}t}\right] \end{array}\;.$$

The evolution of these quantities allows us to simulate the change in the open probability curve in a hair cell recovering from the loss of its tip links using Eq. 4, replacing ${\bar{n}}_{\text{s}}\left(\text{P}\zeta \right)$ and ${\bar{n}}_{\text{p}}\left(\text{P}\zeta \right)$ by *n*_s_(*t*) and *n*_p_(*t*) as given here, respectively. We further simulate the evolution of fast adaptation using Eq. 8, as described above and with the parameters derived from the experiments of (13) on mouse cochlear hair cells. The value of *p*_tip_ is chosen so that the hair bundle has fully recovered by *t* = 48 h. Because the above model reaches *n*_p_ = 50 only asymptotically at *t* = ∞, we consider that full recovery is reached when *n*_p_(*t*) > 49.5, such that the closest integer value is 50. Setting that this value is reached at *t* = 48 h leads to $p_{\text{tip}}≃0.14$. In Fig. 7, we show the resulting open probability curves at 2, 6, 12, 24, 36, and 48 h after tip-link severing.

In the inset, we show the change in the fast-adaptation time constant at the level of the entire hair bundle, following the same fitting procedure as the one used in the previous section. The results agree with those observed experimentally in the mouse cochlea (15, 19); the slope of the open probability curve increases during tip-link regeneration, similarly to the increase during postnatal development (see Fig. 4), whereas adaptation becomes faster, again resembling the change in the kinetics during development (see Fig. 6).

### Adaptation kinetics as a function of the transduction-current amplitude

At intermediate developmental stages, in which both types of units are present, we expect adaptation to become slower with larger imposed displacements. This is because larger displacements open a larger proportion of channels in single-channel units, which adapt more slowly. Such a behavior has been observed experimentally in cochlear hair cells of mice (9, 13, 15) and rats (Fig. 5 E in (14)) several days after birth. In fully mature hair cells, in which we predict that all tip links are connected to the same type of units, this dependency should vanish. In mature hair cells from the mouse utricle and from the frog sacculus, the time constant of fast adaptation is indeed independent of displacement (46, 47). Moreover, according to our model, hair cells with a greater total number of channels have also a greater fraction of them in pairs. Hence, adaptation is expected to be faster for cells that exhibit a larger maximal transduction current. Such a behavior has been reported in Fig. 2 b of (9).

**Figure 8:**
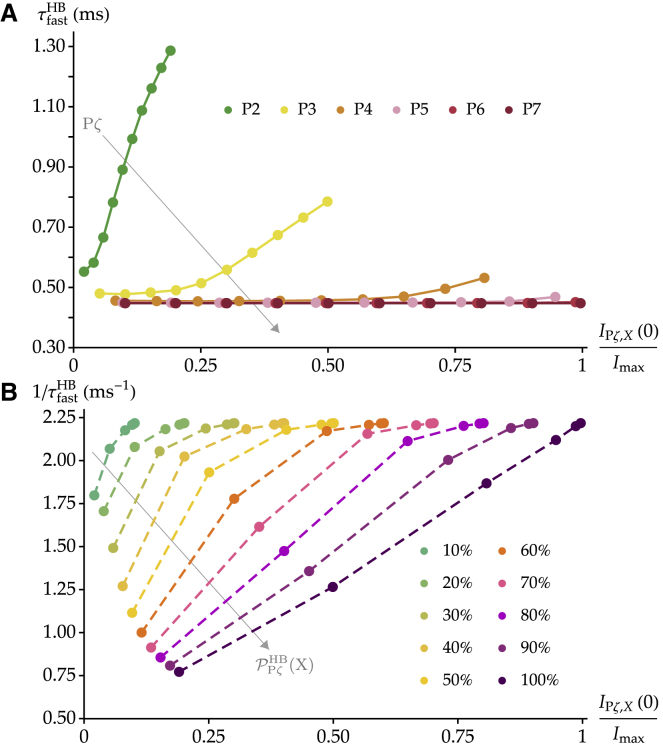
Adaptation kinetics as a function of the normalized peak transduction current at each developmental stage and imposed displacement. (*A*) The time constant of fast adaptation for the whole hair bundle $\tau_{\text{fast}}^{\text{HB}}$ is plotted as a function of the normalized current at *t* = 0 as the displacement *X* is varied at each developmental stage P*ζ* from P2 to P7. The gray arrow indicates the progression toward increasing values of P*ζ* across the different curves. In each series, each data point corresponds to a different value of the overall open probability before adaptation $P_{\text{P}\zeta }^{\text{HB}}\left(X\right)$, as given by Eq. 4, from 10 to 100% in steps of 10%. (*B*) The rate of fast adaptation $1/\tau_{\text{fast}}^{\text{HB}}$ is plotted as a function of the same quantity as in (*A*), this time gathered per equal values of $P_{\text{P}\zeta }^{\text{HB}}\left(X\right)$. The gray arrow indicates the progression toward increasing values of $P_{\text{P}\zeta }^{\text{HB}}\left(X\right)$ across the different curves. The plots are generated with the parameters characterizing the data in Lelli et al. from the mouse cochlea (13), as in Fig. 6, *A* and *B*. To see this figure in color, go online.

In Fig. 8, we investigate the kinetics of fast adaptation at the level of the entire hair bundle as a function of the transduction-current amplitude using Eq. 8 at different developmental stages. In Fig. 8 *A*, we plot the time constant of fast adaptation for the entire hair bundle $\tau_{\text{fast}}^{\text{HB}}$ as a function of *I*_P*ζ*,*X*_(0)/*I*_max_ for each developmental stage P*ζ* between P2 and P7 and imposed displacement *X*. Here, *I*_P*ζ*,*X*_(0) is the negative peak current for a given displacement *X* at the onset of adaptation (t = 0) in a hair cell at the developmental stage P*ζ*, and *I*_max_ = *n*_ch_(P7) × *i*_ch_ is the maximal value of the transduction current at maturation. Note that we exclude from this analysis the developmental stages P0 and P1, for which paired channels are not always present. At each developmental stage P*ζ*, each data point corresponds to a different value of the overall open probability $P_{\text{P}\zeta }^{\text{HB}}\left(X\right)$ at *t* = 0 from 10 to 100% in steps of 10%. We can see that the amplitude of the imposed hair-bundle displacement, directly related to the value of the current *I*_P*ζ*,*X*_(0), has a strong influence on the adaptation kinetics at early developmental stages of the cell and progressively less influence at later developmental stages. At P7, as only paired channels are present, this influence completely disappears. At P2, however, the effective time constant more than doubles between the two extreme values. This trend is in agreement with the results reported in mouse inner hair cells (15) as well as in rat outer hair cells (14).

To compare with other experimental data (9), we plot in Fig. 8 *B* the rate of fast adaptation $1/\tau_{\text{fast}}^{\text{HB}}$ as a function of the same quantity as in Fig. 8 *A*. We group these values per equal open probability, and we can see that, at fixed open probability, the rate of adaptation increases with the peak current across development, in agreement with the experimental results of (9). When using parameters derived from the data in Waguespack et al. (14) and Eq. 11, this analysis yields similar results (see Fig. S4).

## Discussion

The maturation of mechanotransduction in cochlear hair cells across several days during postnatal development, as well as across several hours during tip-link regeneration, is characterized by a number of hitherto unexplained variations in the MET currents’ biophysical properties. Instead of relying on the differential expression of molecularly distinct MET channels, we have explained these changes as the result of a random connection of tip links to a population of MET channels that individually have constant biophysical properties. In this framework, the maturation of the MET current’s biophysical properties is the result of a change in the relative numbers of transduction units that contain either a single or multiple channels. Although composed of the same kind of channels, these two types of units behave differently because clustered channels display a cooperative behavior that enhances their mechanosensitivity. Here, we quantitatively explored the case of the model developed in (30), in which mature transduction units comprise pairs of channels that gate cooperatively because of their interaction via the membrane bilayer. In the model presented here, single channels display relatively broad open probability curves, centered at relatively large hair-bundle displacements, whereas paired channels display steeper open probability curves, centered at smaller displacements. In general, as long as these qualitative differences between the open probability curves exist, this analysis holds true regardless of the specific mechanism of cooperativity between MET channels.

Using this framework, we have reproduced the following aspects of hair-cell postnatal development. First, as development proceeds, hair cells become more sensitive in that the channels gate at smaller hair-bundle displacements and over a narrower range of displacements. Second, at intermediate developmental stages, the transduction current versus displacement curves have an asymmetric shape, increasing more rapidly in the first half of their gating range. Third, the time constants of fast and slow adaptation at the level of the entire hair bundle decrease during development. Fourth, at intermediate developmental stages, the time constant of fast adaptation depends on the amplitude of the imposed hair-bundle displacement. In addition, we showed that we could reproduce the similar biophysical changes observed during tip-link regeneration using the same model.

To reproduce the change in the measured fast adaptation kinetics, we hypothesized that the adaptation time constants for single and paired channels are different. Published ensemble-averaged traces based on single-channel recordings suggest that single channels do not always adapt (Fig. 5 in (48)). More specifically, ensemble-averaged current traces in a hair cell from the apex of the mouse cochlea at P2 show no adaptation. As these recordings represent the activity of a MET unit whose conductance is at the lower end of the observed range, they are most likely due to the activity of a single channel. In contrast, prominent adaptation has been observed in ensemble-averaged traces from relatively more mature basal hair cells at P2 and apical hair cells at P4 (29). The associated conductance values sit in the middle or at the upper end of the measured range, respectively, which, within our framework, indicates that they reflect the concerted opening of more than one channel per MET unit, as suggested by the authors of that study. Another series of similar recordings from mouse outer hair cells at P4 and P6 showed current traces with several peaks in the amplitude histograms, interpreted as the concerted openings of different numbers of MET channels (29). Those records showed a prominent adaptation as well.

In the model developed in (30) and used here, energy is required not only to open the paired channels but also to close them. This is because, when the channels are in contact, the state corresponding to both channels open is stable independently of tip-link tension because of the favorable membrane-mediated interaction. The energy required to escape from this configuration and close the two channels can be estimated from the sum of the following contributions. The energy difference of one adaptation spring between the closed and open states of the channel is [(1/2)*k*_a_(*e*_a,max_)^2^ − (1/2)*k*_a_(*e*_a,max_ − *δ*)^2^] in favor of the open state, where *e*_a,max_ is the extension of the adaptation springs when the channels touch in the CC configuration, *δ* is the variation of the channel lateral width upon gating, and *k*_a_ is the stiffness of the adaptation springs. With the default parameters reported in Table S2, we have $e_{\text{a},\text{max}}≃12$ nm (see (30)), which leads to an energy difference of 5.4 *k*_B_*T* in favor of the open state. With the contribution from the channel gating energy *E*_g_ = 9 *k*_B_*T*, we obtain a total contribution of 3.6 *k*_B_*T* per channel in favor of the closed state or 7.2 *k*_B_*T* for the pair. Finally, the contribution from the membrane elastic energies is a difference of 22.5 *k*_B_*T* in favor of the OO configuration versus the CC one. Altogether, the OO state is therefore favored by ∼15.3 *k*_B_*T* over the CC state at the point at which the paired channels are in contact. With two channels per tip link, this corresponds to an energy of ∼7.6 *k*_B_*T* per channel and per cycle. This value is similar to the approximate 7 *k*_B_*T* per single Ca^2+^ binding-unbinding event (49). After multiplication by a total of 100 channels in our model of a fully developed hair bundle, an estimated total input energy of ∼700–800 *k*_B_*T* is required to power mechanotransduction.

This result has two broader implications. First, mechanotransduction in a fully developed hair bundle requires an external energy source. Second, mechanotransduction and fast adaptation are both active processes, relying on the same external energy source, potentially the out-of-equilibrium gradient of Ca^2+^ concentration across the cell membrane (49). Thanks to such an active process, the paired channels would close and the adaptation springs would separate them, in turn affecting tip-link tension and ultimately performing mechanical work on the hair bundle on a cycle-by-cycle basis. Other passive properties related to fast adaptation, such as tip-link viscoelasticity (50, 51), could coexist with this active process. However, they cannot replace it. In contrast, single channels do not require energy to close but can do so passively whenever tip-link tension is reduced. This allows the transduction current to passively follow the external stimulus on a cycle-by-cycle basis. Physiologically, this property could be beneficial for hair-cell development, which requires some resting MET current to progress normally (52). As the MET machinery fully matures and single-channel units are replaced by paired-channel ones, the active process would progressively come into play, and mechanotransduction and amplification become intimately linked.

## Author Contributions

F.G. designed and developed the theory, performed the calculations and the simulations, produced the figures, interpreted the results, and wrote the manuscript. T.R. supervised the development of the theory, interpreted the results, and wrote the manuscript. A.S.K. conceived the proposed mechanism, designed and supervised the project, interpreted the results, and wrote the manuscript. All authors contributed extensively to this work.

## Supporting Citations

References (53, 54, 55, 56, 57, 58) appear in the Supporting Material.

## Supporting Material

Document S1. Supporting Material, Figs. S1–S4, and Tables S1–S2

Document S2. Article plus Supporting Material
