# Supplementary material for: The Development of Cooperative Channels Explains the Maturation of Hair Cell’s Mechanotransduction
Source: Biophys J. 2019 Sep 12;117(8):1536–48. doi: 10.1016/j.bpj.2019.08.042 (PMC6817549; doi:10.1016/j.bpj.2019.08.042)
Supplement: Document S1. Supporting Material, Figs. S1–S4, and Tables S1–S2 [file mmc1.pdf]

Biophysical Journal, Volume *117*

Supplemental Information

The Development of Cooperative Channels Explains the Maturation of Hair Cell's Mechanotransduction

Francesco Gianoli, Thomas Risler, and Andrei S. Kozlov

The development of cooperative channels explains the maturation of hair cell's mechanotransduction – Supporting Material –

Francesco Gianoli[1], Thomas Risler[2,*], and Andrei S. Kozlov[1,*]

[1]Department of Bioengineering, Imperial College London, London SW7 2AZ, United Kingdom
[2]Laboratoire Physico-Chimie Curie, Institut Curie, PSL Research University, Sorbonne Université, CNRS, 26 rue d'Ulm, 75005 Paris, France
[*]Correspondence: a.kozlov@imperial.ac.uk or thomas.risler@curie.fr

LINEAR AND SIGMOIDAL GROWTHS IN THE NUMBER OF CHANNELS

Population growth of MET channels

	Linear				Sigmoidal			
Maturation	P6		P7		P6		P7	
	n_{ch}	$\bar{n}_p$	n_{ch}	$\bar{n}_p$	n_{ch}	$\bar{n}_p$	n_{ch}	$\bar{n}_p$
P0	0	0	0	0	1	0	1	0
P1	17	1	14	1	5	0	4	0
P2	33	5	29	4	19	2	13	1
P3	50	12	43	9	50	12	35	6
P4	67	22	57	16	81	33	65	21
P5	83	34	71	25	95	45	87	38
P6	100	50	86	35	99	49	96	46
P7	100	50	100	50	100	50	99	49

Table 1: Number of channels n_{ch} and associated most-likely number of channel pairs $\bar{n}_p$ in a hair bundle with 50 tip links, corresponding to $n_{max} = 100$ channels, as a function of the developmental stage Pζ. Two different types of growths of the total number of channels are reported: a linear and a sigmoidal growths. For each of these growth models, we simulate the development over a total maturation time ζ_{max} of either six or seven days, to account for the variations observed experimentally. The sigmoidal growth is dictated by Eq. 1 of the main text with $\bar{\zeta} = \zeta_{max}/2$ and $\nu = 1.26$ or 1.46 per day, respectively for these two cases. For each of the four obtained population growths, the most likely number of channel pairs $\bar{n}_p$ is given by the maximization of Eq. 3 of the main text at fixed n_{ch} and n_t, rounded up to the closest integer value.

PARAMETERS OF THE MODEL

Table 2: List of parameters

Parameters characterizing the hair bundle and the mechanotransduction units

Par.	Description		Default value	Unit
k_t	Tip link's stiffness	Tobin 2018 (1)	0.7	$mN{\cdot}m^{-1}$
δ	Channel's steric change upon gating	Ursell 2007 (2)	2	nm
γ	Geometrical projection factor	Tobin 2018 (1)	0.1	adim.
E_g	Channel's gating energy	Corey 1983 (3); Hudspeth 1992 (4); Ricci 2006 (5)	9	k_BT
N	Number of tip links	Howard 1988 (6); Tobin 2018 (1)	50	adim.
K_{sp}	Combined stiffness of the stereociliary pivots	Tobin 2018 (1)	2.1	$mN{\cdot}m^{-1}$
l	Length of the tip link's branch	Kachar 2000 (7), Araya-Secchi 2016 (8)	13	nm
k_a	Adaptation spring's stiffness	Powers 2012 (9)	1	$mN{\cdot}m^{-1}$
α	Angle of the adaptation springs w.r.t the horizontal	Kachar 2000 (7); Powers 2012 (9)	5	degrees
a_{adapt}	Value of a with relaxed adaptation springs (CC config.)	Kachar 2000 (7)	13	nm
ρ	Radius of the closed channel	Ursell 2007 (2)	2.5	nm
a_{min}	Minimum value of a	Ursell 2007 (2)	1.25	nm
X_{sp}	Resting position of the pivots	Tobin 2018 (1)	21	nm
$X_{1/2}$	Value of the hair-bundle displacement X such that $\mathcal{P}_s(X) = 0.5$	Howard 1988 (6)	118	nm

Parameters characterizing the elastic membrane potentials

Par.	Description		Default value	Unit
$a_{cross,CC}$	Crossing point of the CC potential	All parameters in this section are from Ursell 2007 (2)	3	nm
$a_{cross,OC}$	Crossing point of the OC potential		2.75	nm
$a_{cross,OO}$	Crossing point of the OO potential		2.5	nm
E_{CC}	Value of the CC potential at a_{min}		−2.5	k_BT
E_{OC}	Energy scale of the OC potential		50	k_BT
E_{OO}	Value of the OO potential at a_{min}		−25	k_BT
l_V	Potentials' decay length		1.5	nm

OPEN PROBABILITY AND ITS DERIVATIVE WITH A LINEAR GROWTH OF THE CHANNEL POPULATION ACROSS DEVELOPMENT

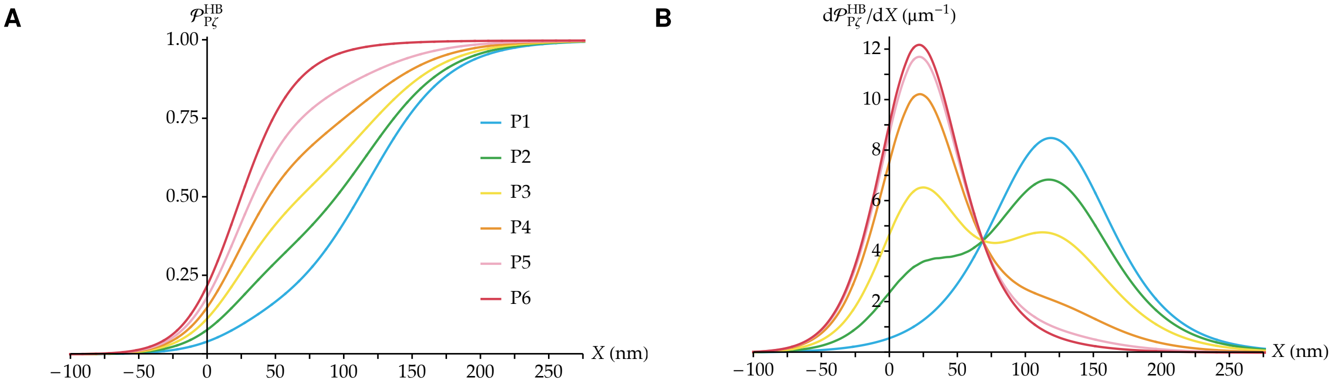

Figure 1: (A) Simulated maturation of the open-probability vs. displacement curve $\mathcal{P}^{HB}_{P\zeta}(X)$ in a developing hair cell. We report the case of a linear growth of the number of MET channels over six days, as given in Table S1. The parameters that define the transduction units are the same as in Fig. 3 of the main text. (B) Derivatives of the open probability curves of panel A, using the same color code.

MATURATION OF SLOW ADAPTATION

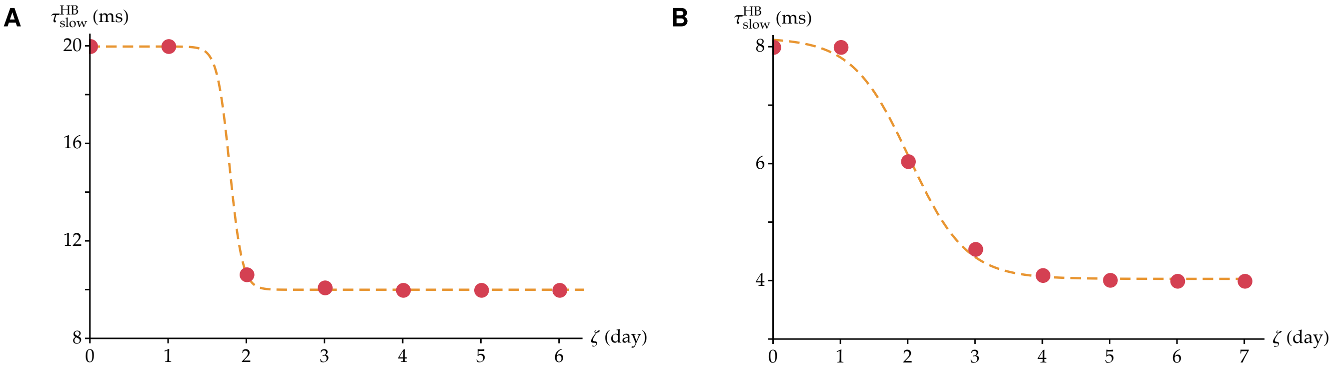

Figure 2: Time constant of slow adaptation τ_{slow}^{HB} for the entire simulated hair bundle associated with the results reported in Fig. 6. (*A*) Slow adaptation time constant associated with Fig. 6A. (*B*) Slow adaptation time constant associated with Fig. 6C.

EFFECT OF A SHIFT IN THE POPULATION GROWTH ON FAST ADAPTATION

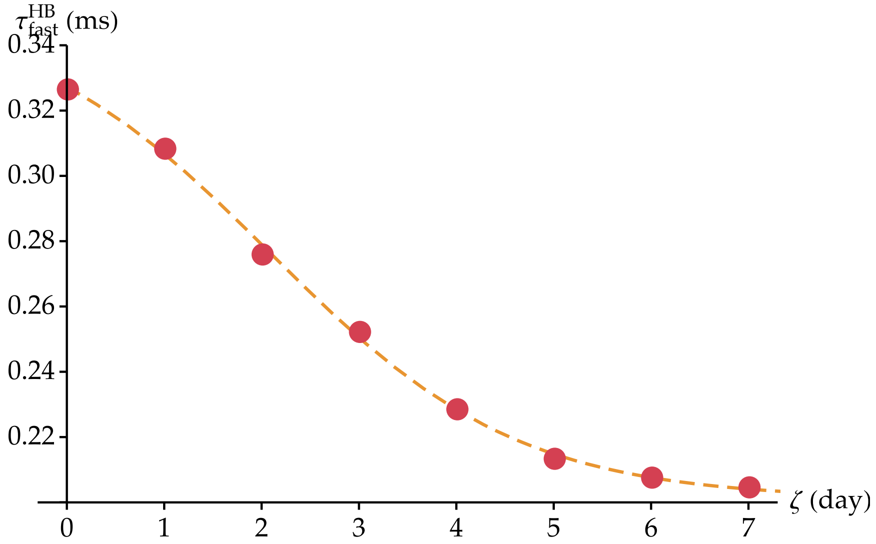

Figure 3: Simulated maturation of the time constant of fast adaptation τ_{fast}^{HB} for the entire hair bundle as a function of developmental stages, assuming a sigmoidal growth in the number of channels over ten days, where the population growth has been shifted by three days to reach maturation at P7. Parameters are the same as in Fig. 6*C*,*D*. A sigmoidal fit is used to visualize the trend.

ADAPTATION KINETICS AS A FUNCTION OF THE TRANSDUCTION-CURRENT AMPLITUDE

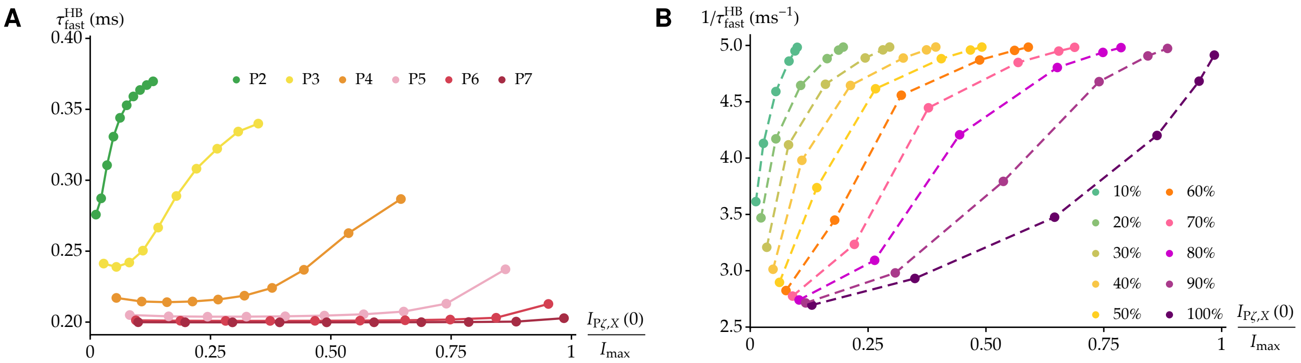

Figure 4: Adaptation kinetics as a function of the normalized peak transduction current at each developmental stage and imposed displacement, using the parameters determined from the data in Waguespack *et al.* (10) and Eq. 11 of the main text. (A) The time constant of fast adaptation for the whole hair bundle $\tau_{\rm fast}^{\rm HB}$ is plotted as a function of the normalized current at $t = 0$ as the displacement X is varied, at each developmental stage Pζ from P2 to P7. In each series, each data point corresponds to a different value of the overall open probability before adaptation $\mathcal{P}_{\rm P\zeta}^{\rm HB}(X)$, as given by Eq. 4 of the main text, from 10% to 100% in steps of 10%. (B) The rate of fast adaptation $1/\tau_{\rm fast}^{\rm HB}$ is plotted as a function of the same quantity as in (A), this time gathered per equal values of $\mathcal{P}_{\rm P\zeta}^{\rm HB}(X)$. The plots are generated with the parameters characterizing the data in Waguespack *et al.* from the rat cochlea (10), as in Fig. 6C,D of the main text.

COMPUTATION OF $X_{1/2}$ IN THE GATING-SPRING MODEL

Within the standard gating-spring model (6), force balance at the level of the hair bundle reads (see Eq. 10 in (6)):

$$F = K_{\rm sp}(X - X_{\rm sp}) + N\kappa_{\rm G}\gamma\left(\gamma X + x_{\rm r} + \frac{\delta}{2}\right) - Nz\mathcal{P}_{\rm s}(X)\,, \tag{1}$$

where F is the steady-state force required to hold the hair bundle at position X, $X_{\rm sp}$ is the resting position of the stereociliary pivots, $\kappa_{\rm G}$ is the gating-spring stiffness, $x_{\rm r}$ is the gating spring's resting extension when the hair bundle is unperturbed, and all other parameters have been introduced in the main text and are summarized in Table S2. With our notation, Eqs. 6 and 8 of ref. (6) lead to $z = \gamma k_{\rm t}\delta$, as already reported in the main text, and $x_{\rm r} = (E_{\rm g} - zX_{1/2})/(\kappa_{\rm G}\delta)$. Imposing that the hair bundle sits at $X = 0$ when $F = 0$, we get, with $\kappa_{\rm G} = k_{\rm t}$:

$$\frac{\gamma}{\delta}(E_{\rm g} - zX_{1/2}) - \frac{z}{1 + \exp[zX_{1/2}/(k_{\rm B}T)]} + \frac{1}{2}\gamma k_{\rm t}\delta - \frac{K_{\rm sp}}{N}X_{\rm sp} = 0\,, \tag{2}$$

which specifies the value of $X_{1/2}$.

SUPPORTING REFERENCES

1. Tobin, M., A. Chaiyasitdhi, V. Michel, N. Michalski, and P. Martin, 2019. Stiffness and tension gradients of the hair cell's tip-link complex in the mammalian cochlea. *eLife* 8:e43473. https://elifesciences.org/articles/43473.

2. Ursell, T., K. C. Huang, E. Peterson, and R. Phillips, 2007. Cooperative gating and spatial organization of membrane proteins through elastic interactions. *PLOS Comput. Biol.* 3:e81. http://journals.plos.org/ploscompbiol/article?id=10.1371/journal.pcbi.0030081.

3. Corey, D. P., and A. J. Hudspeth, 1983. Analysis of the microphonic potential of the bullfrog's sacculus. *J. Neurosci.* 3:942–961. http://www.jneurosci.org/content/3/5/942.

4. Hudspeth, A. J., 1992. Hair-bundle mechanics and a model for mechanoelectrical transduction by hair cells. *Soc. Gen. Physiol. Ser.* 47:357–370. https://www.ncbi.nlm.nih.gov/pubmed/1369770.

5. Ricci, A. J., B. Kachar, J. Gale, and S. M. Van Netten, 2006. Mechano-electrical transduction: New insights into old ideas. *J. Membr. Biol.* 209:71–88. https://link.springer.com/article/10.1007/s00232-005-0834-8.

6. Howard, J., and A. J. Hudspeth, 1988. Compliance of the hair bundle associated with gating of mechanoelectrical transduction channels in the Bullfrog's saccular hair cell. *Neuron* 1:189–199. https://www.sciencedirect.com/science/article/abs/pii/0896627388901390.

7. Kachar, B., M. Parakkal, M. Kurc, Y.-d. Zhao, and P. G. Gillespie, 2000. High-resolution structure of hair-cell tip links. *Proc. Natl. Acad. Sci. U.S.A.* 97:13336–13341. https://www.pnas.org/content/97/24/13336.short.

8. Araya-Secchi, R., B. L. Neel, and M. Sotomayor, 2016. An Elastic Element in the Protocadherin-15 Tip Link of the Inner Ear. *Nat. Commun.* 7:13458. https://www.nature.com/articles/ncomms13458.

9. Powers, R. J., S. Roy, E. Atilgan, W. E. Brownell, S. X. Sun, P. G. Gillespie, and A. A. Spector, 2012. Stereocilia membrane deformation: Implications for the gating spring and mechanotransduction channel. *Biophys. J.* 102:201–210. https://www.sciencedirect.com/science/article/pii/S0006349511054245.

10. Waguespack, J., F. T. Salles, B. Kachar, and A. J. Ricci, 2007. Stepwise Morphological and Functional Maturation of Mechanotransduction in Rat Outer Hair Cells. *J. Neurosci.* 27:13890–13902. https://www.jneurosci.org/content/27/50/13890.short.
